# Reconstruction of the ancestral marsupial karyotype from comparative gene maps

**DOI:** 10.1186/1471-2148-13-258

**Published:** 2013-11-21

**Authors:** Janine E Deakin, Margaret L Delbridge, Edda Koina, Nerida Harley, Amber E Alsop, Chenwei Wang, Vidushi S Patel, Jennifer A Marshall Graves

**Affiliations:** 1 ARC Centre of Excellence for Kangaroo Genomics, Canberra, Australia; 2 Evolution, Ecology and Genetics; Research School of Biology, The Australian National University, Canberra ACT 0200, Australia; 3 Institute of Applied Ecology, University of Canberra, Canberra ACT 2601, Australia; 4 Current Address: Cytogenetics Department, ACT Pathology, The Canberra Hospital, Yamba Drive, Canberra 2605, Australia; 5 Current Address: Surgery, The University of Western Australia, 35 Stirling Highway, Crawley WA 6009, Australia; 6 Current Address: Walter & Eliza Hall Institute of Medical Research, 1G Royal Parade, Parkville, Victoria 3052, Australia; 7 Australian Prostate Cancer Research Centre-Queensland, Queensland University of Technology, 199 Ipswich Rd, Brisbane, QLD 4102, Australia; 8 La Trobe Institute of Molecular Sciences, La Trobe University, Melbourne, Victoria 3086, Australia

**Keywords:** Comparative genomics, Comparative mapping, Physical map, Marsupial, Ancestral karyotype

## Abstract

**Background:**

The increasing number of assembled mammalian genomes makes it possible to compare genome organisation across mammalian lineages and reconstruct chromosomes of the ancestral marsupial and therian (marsupial and eutherian) mammals. However, the reconstruction of ancestral genomes requires genome assemblies to be anchored to chromosomes. The recently sequenced tammar wallaby (*Macropus eugenii*) genome was assembled into over 300,000 contigs. We previously devised an efficient strategy for mapping large evolutionarily conserved blocks in non-model mammals, and applied this to determine the arrangement of conserved blocks on all wallaby chromosomes, thereby permitting comparative maps to be constructed and resolve the long debated issue between a 2n = 14 and 2n = 22 ancestral marsupial karyotype.

**Results:**

We identified large blocks of genes conserved between human and opossum, and mapped genes corresponding to the ends of these blocks by fluorescence in situ hybridization (FISH). A total of 242 genes was assigned to wallaby chromosomes in the present study, bringing the total number of genes mapped to 554 and making it the most densely cytogenetically mapped marsupial genome. We used these gene assignments to construct comparative maps between wallaby and opossum, which uncovered many intrachromosomal rearrangements, particularly for genes found on wallaby chromosomes X and 3. Expanding comparisons to include chicken and human permitted the putative ancestral marsupial (2n = 14) and therian mammal (2n = 19) karyotypes to be reconstructed.

**Conclusions:**

Our physical mapping data for the tammar wallaby has uncovered the events shaping marsupial genomes and enabled us to predict the ancestral marsupial karyotype, supporting a 2n = 14 ancestor. Futhermore, our predicted therian ancestral karyotype has helped to understand the evolution of the ancestral eutherian genome.

## Background

Metatherians (marsupials) are a diverse group of mammals found in the Americas and Australasia. They diverged from eutherian (“placental”) mammals approximately 143-178 million years ago (MYA) [1,2] and possess many unique biological features that have intrigued biologists since these animals were first described. The American and Australian superorders (Ameridelphia and Australidelphia) diverged about 80MYA, and it is generally acknowledged that the earliest offshoots of the marsupial lineage were the families Didelphidae and Caenolestidae that colonised the Americas, and that later offshoots gave rise to the Australian expansion [3,4].

One feature of marsupials that has been extensively studied over the past 100 years is their chromosomes. Their characteristically large chromosomes and low diploid numbers have made marsupial chromosomes easy to study, and the karyotypes of approximately 70% species have been determined [5]. Studies of marsupial chromosome number, morphology and G-banding revealed an astonishing level of conservation across the entire infraclass of Metatheria. This was supported by cross-species chromosome painting, which demonstrated that all karyotypic variation amongst marsupials could be attributed to the arrangement of just 19 conserved segments [6].

Two diploid numbers predominate amongst marsupials, with 2n = 14 complements found in six of seven extant marsupial orders and 2n = 22 also common amongst both American and Australian species. The prevalence of these two diploid numbers led to two alternative hypotheses for the ancestral marsupial chromosome number. The first posits a marsupial ancestor with a 2n = 14 karyotype, a chromosome complement observed to have changed little between divergent species [7], with fissions giving rise to higher diploid numbers that are seen in many families [7-10]. The alternative hypothesis proposes that the 2n = 14 karyotype common to many species is derived from fusion events from an ancestor with 22 chromosomes [11,12].

Debate over which of these hypotheses is more likely has continued for almost 40 years with varying levels of support provided for each one. Most evidence supports a 2n = 14 ancestor, with very similar G-banding patterns observed between species with a 2n = 14 karyotype and any differences in chromosome morphology accounted for by inversions or intrachromosomal rearrangements [7]. Westerman et al. [13] used cytogenetic information plotted onto a phylogenetic tree to provide additional support for the 2n = 14 ancestral karyotype, with basal species on this well-resolved phylogenetic tree possessing a 2n = 14 karyotype. The conserved segment composition, determined by chromosome painting, in species with an ‘ancestral’ 2n = 14 chromosome complement is the same across the different families of marsupials, although the arrangement of the segments on individual chromosomes may vary due to intrachromosomal rearrangements [6,14,15]. The derivations of other chromosome complements from this predicted ancestral arrangement, mainly by fission events, have been proposed [13,16].

Evidence for the alternative hypothesis of 2n = 22 relies on the frequency of this diploid number, particularly in the Ameridelphidae, and on evidence for chromosome fusions. The 2n = 22 chromosome complement is common amongst marsupials; however, the arrangement of the 18 conserved autosomal segments is not the same between 2n = 22 species from different families [6,17], weakening the argument for it being the ancestral chromosome number. If the marsupial ancestor had a 2n = 22 chromosome complement, it would probably resemble karyotypes of members of Family Didelphidae with 22 chromosomes, since the American marsupials are at the base of the marsupial phylogenetic tree [13,18]. The strongest evidence for the higher ancestral number is the presence of interstitial telomere signals in members of Didelphidae with 2n = 18 or 2n = 14 karyotypes, suggesting that lower diploid number karyotypes were derived by chromosome fusions, ultimately leading to the 2n = 14 karyotype commonly found amongst marsupials, and recognised as the basal karyotype of Australidelphia [12,19]. However, Pagnozzi et al. [20,21] observed that these interstitial signals coincide with constitutive heterochromatin, and proposed that they actually represent satellite DNA rather than telomeric sequence, as was also concluded for an Australian species with a 2n = 14 karyotype [22]. However, Svartman [23] argued that at least for the grey slender opossum (*Marmosops incanus*), interstitial signals fall outside the region of pericentromeric heterochromatin, leaving the issue of the ancestral marsupial chromosome complement still open for debate.

Resolving the ancestral karyotype has been difficult without the detailed information that permits reference to an outgroup. This becomes possible now that three marsupial genomes have been sequenced; one representing the American clade (grey short-tailed South American opossum) and two representing distantly related Australian marsupials (tammar wallaby and Tasmanian devil, the last having the 2n = 14 karyotype predicted to be ancestral at least to Australidelphia). Comparing the arrangement of genes on chromosomes between these species and with outgroups such as chicken and human could help reconstruct the karyotype of the marsupial ancestor.

The South American opossum (*Monodelphis domestica*) genome assembly, constructed from an almost 7-fold coverage of Sanger sequencing, has 97% of its sequence anchored to eight autosomes and the X chromosome [24,25]. The Tasmanian devil genome has been sequenced entirely by next generation sequencing technology but the sequence has not been ordered on chromosomes [26,27]. A physical map of the devil genome has been constructed with 105 BACs mapped to chromosomes [28], but this map is not sufficiently dense to accurately reconstruct an ancestral karyotype. The tammar wallaby (*Macropus eugenii*) genome assembly from 2-fold Sanger sequencing coverage is highly fragmented and assignment of the 379,858 [29] sequence scaffolds to its seven autosomes and X chromosome using the same approach used for the opossum genome would be an arduous task. Determining how the sequence is arranged on chromosomes is imperative for reconstruction of an ancestral karyotype.

In order to reconstruct the most likely ancestral marsupial karyotype, we therefore constructed a dense physical map of the wallaby genome. To accomplish this task efficiently, we employed the strategy devised to construct a physical and virtual map of two wallaby chromosomes [30] to map other autosomes. We then combined this mapping data with previously published data to produce a map of the entire wallaby genome. Comparisons of the wallaby map to the opossum genome assembly facilitated the delineation of the conserved segment boundaries identified by chromosome painting, and permitted the detection of rearrangements undetected by previous G-banding or chromosome painting.

By comparing the wallaby map to opossum, and these marsupial maps with chicken and eutherian species, we were able to determine the ancestral arrangement of the 19 conserved segments, and gain insight into the arrangement of conserved gene blocks in the ancestor of therian (marsupial and eutherian) mammals. This comparative mapping data provides strong support for a marsupial ancestor with a smaller rather than larger diploid number.

## Results and discussion

Reconstruction of the ancestral marsupial karyotype firstly required construction of a map of the wallaby genome, so that comparisons of gene arrangement between the wallaby and opossum genomes could be made. We used the strategy originally devised to construct a physical and virtual map of wallaby chromosome 5, which identified conserved blocks of genes that are syntenic in opossum and human, and mapped the ends of these blocks by FISH to wallaby chromosomes [30]. Our analysis enabled us to reconstruct the karyotype of the marsupial ancestor, and also provided insight into the genome organisation of the therian ancestor.

### Cytogenetic map of the tammar wallaby genome

We identified 154 conserved blocks of genes that shared synteny in both opossum and human genomes using Ensembl synteny viewer [31]. These were taken from six of the eight opossum autosomes, since cytogenetic maps had previously been constructed for wallaby chromosomes 5 and 6q (corresponding to opossum chromosomes 4 and 7 respectively) using the strategy outlined above [30,32]. The average block size based on the opossum genome assembly was 16.2 Mb, ranging from the largest block (218 Mb) on opossum chromosome 5 to the smallest (30 kb) on opossum chromosome 6. Not surprisingly, chromosome 1 (spanning 749 Mb) contained the most blocks (48), but chromosome 6 (spanning only 292 Mb) contained 38 blocks (Table 1).

**Table 1:** Conserved block details for each opossum chromosome

**Chromosome**	**Number of opossum-human conserved blocks**	**Smallest block (Mb)**	**Largest block (Mb)**	**Average block size (Mb)**
1	48	0.2	83	15
2	22	0.3	168	24
3	22	0.07	82	23
4	18^1^	0.7	117	117
5	5	1.6	218	60.3
6	38	0.03	48	7.4
7	12^2^	0.2	95	21.2
8	19	1	87	15.5
X	24^1^	0.14	9.7	1.9
Overall	208	0.03	218	16.2

^1^Blocks mapped in Deakin et al. [30].

^2^Blocks were mapped by Deakin et al. [30] and Mohammadi et al. [32].

Gene order between opossum and human was conserved within many of these blocks, but within some blocks, genes from the same human chromosome were rearranged by one or more inversions. Because our analysis did not limit the identification of blocks to those with conserved gene order between these two species, we identified considerably fewer, and larger, conserved blocks than the 616 reported previously that had conserved gene order between opossum and human [24]. These larger blocks were more useful for efficient mapping.

**Figure 1:**
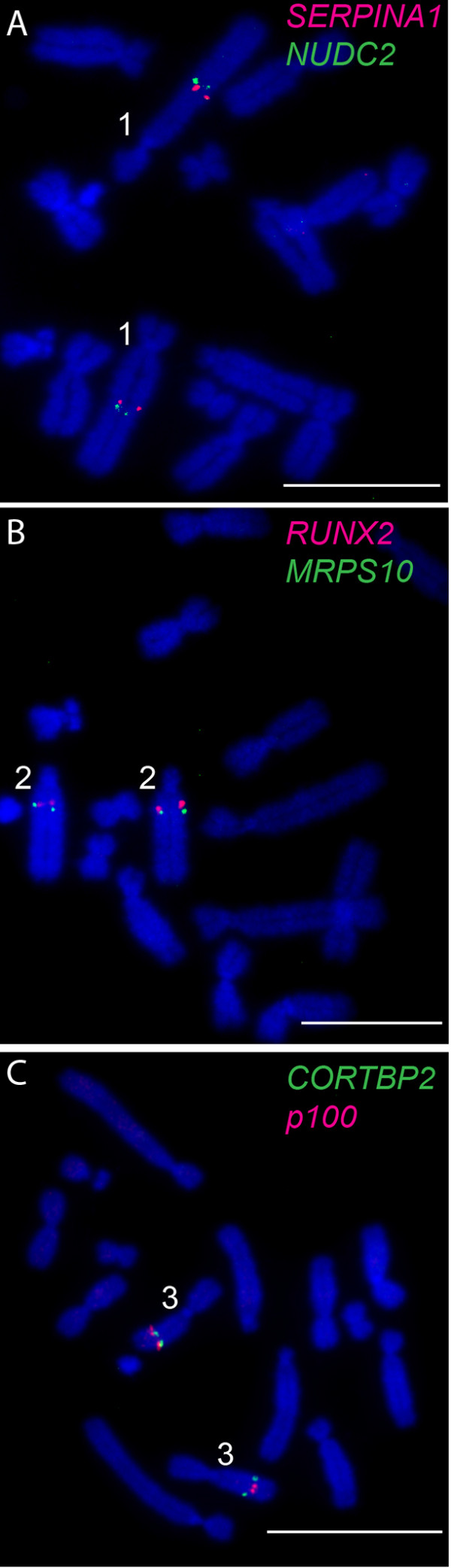
**Examples of FISH determining the orientation of adjacent BAC clones on tammar wallaby metaphase chromosomes.** Orientation of **(A)***SERPINA1* labelled green and *NUDC2* in red on chromosome 1; **(B)***RUNX2* in red and *MRPS10* in green on chromosome 2 and **(C)***CORTBP2* in green and *p100* in red on chromosome 3. Chromosomes have been counterstained with DAPI. Scale bar represents 10 μm.

**Figure 2:**
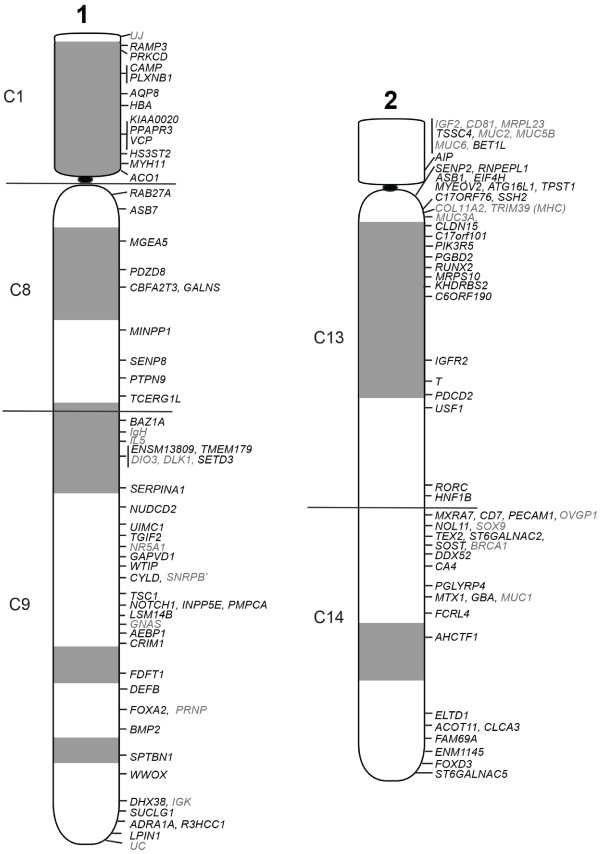
**Cytogenetic map of tammar wallaby chromosomes 1 and 2.** The cytogenetic location of each gene mapped by FISH is indicated alongside the DAPI-banded ideograms. Gene names indicated in grey were mapped as part of previous studies. The boundaries of the conserved segments determined by chromosome painting are indicated by horizontal lines.

**Figure 3:**
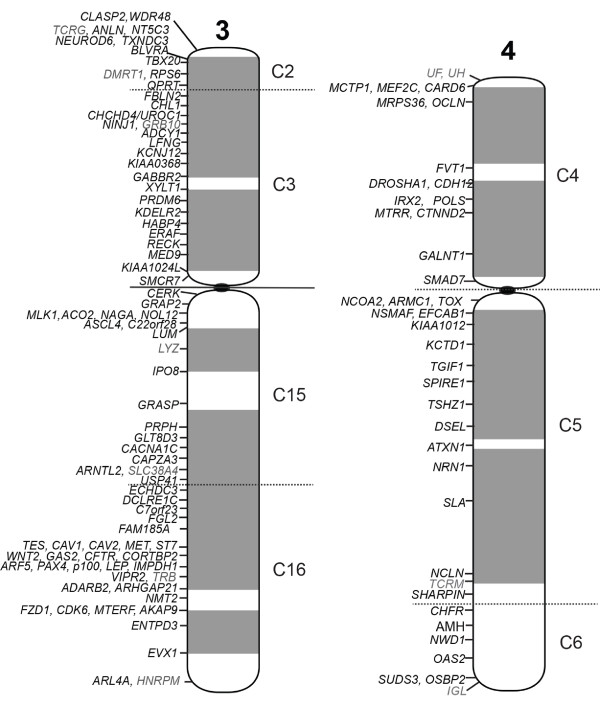
**Cytogenetic map of tammar wallaby chromosomes 3 and 4.** The boundaries of the conserved segments determined by chromosome painting are indicated by horizontal lines; solid lines indicate definitively determined boundaries from wallaby/opossum comparisons and dotted lines represent boundaries which could not be clearly established.

**Figure 4:**
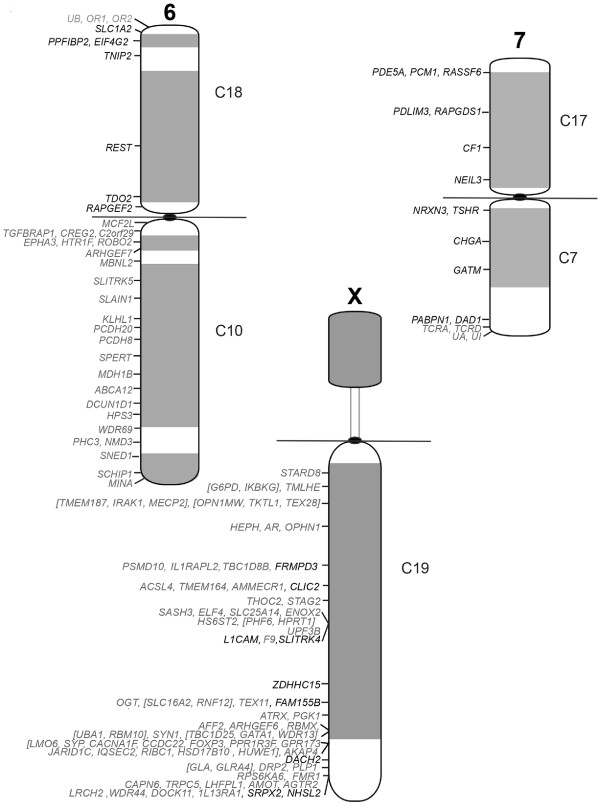
Cytogenetic map of tammar wallaby chromosomes 6, 7 and X.

Given the resolution limitations of FISH on condensed metaphase chromosomes to regions separated by more than 1 Mb [33], we targeted genes at both ends of large conserved blocks (>3 Mb) and one gene within a smaller block (<3 Mb). Wallaby-specific overgo probes were designed for these genes using wallaby genome sequence, and used to screen the wallaby BAC library. BACs containing these genes were mapped using fluorescence in situ hybridisation (FISH). The relative order of genes on the same chromosome was determined by labelling adjacent BACs with different fluorochromes (see Figure 1 for examples). We mapped 242 genes to wallaby chromosomes in this study and combined this with previously obtained physical mapping data (Table 2) to bring the total number of genes assigned to chromosomes in the wallaby to 554 (Figures 2, 3 and 4). The genes mapped and their corresponding BACs are listed in Additional file 1.

**Table 2:** Number of genes mapped to wallaby chromosomes

**Chromosome**	Predicted size* (Mb)	**No. of genes mapped in current study**	**Previously mapped genes**	**Total**
1	486	54	11	65
2	367	47	44	91
3	355	76	7	83
4	340	36	4	40
5	340	0	141	141
6	286	7	28	35
7	133	13	4	17
X	150	9	73	82
Total	2457	242	312	554

*Size predicted from flow sorted chromosomes [38].

Most genes mapped to the chromosome and region predicted from the opossum genome assembly and cross-species chromosome painting data. However, gene order gave information about intrachromosomal rearrangements that are invisible to chromosome painting.

**Figure 5:**
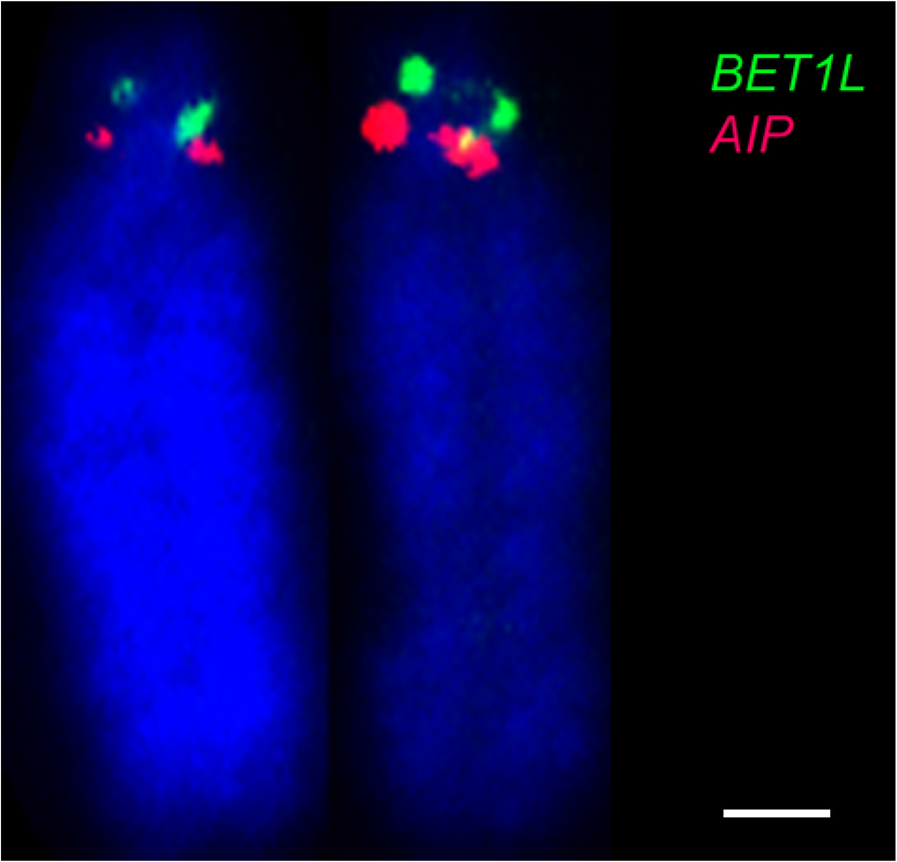
**Mapping of genes to the short arm of wallaby chromosome 2.** FISH mapping of *BET1L* (green) and *AIP* (red) indicates homology to human 11p. Scale bar represents 1 μm.

Gene mapping also resolved previous blank spots on the map where homology between species was not previously identified by chromosome painting and unassigned genes in the opossum genome assembly. For instance, chromosome painting failed to identify the opossum region homologous to wallaby 2p [6]. Several human chromosome 11p15.5 genes (*IGF2, MRPL23, CD81*) had been assigned to wallaby 2p as part of studies into the location of imprinted gene clusters in the wallaby [34,35]. These genes have no chromosomal assignment in the opossum genome assembly [24]. However, *IGF2* has been localised by FISH to opossum 5q3 [36], suggesting that wallaby 2p is homologous to a small region on opossum 5q3. We mapped two other genes (*BET1L, TSSC4*) from human 11p15.5 in the wallaby to 2p, providing more support for this claim. An additional gene (*AIP* from a different region of human chromosome 11q13.3), expected from its opossum location to map to wallaby chromosome 3, also localised to wallaby 2p (Figure 5). This suggests either that a transposition event occurred or there is an error in the opossum genome assembly.

The tammar wallaby cytogenetic map provides a good framework for anchoring genome sequence to chromosomes, which is essential for evolutionary and comparative genome analysis [37]. Our approach of mapping the ends of conserved blocks means that many of the 379,858 sequence scaffolds can be assigned to chromosomes [38]. With 554 genes physically localised to chromosomes, the wallaby represents the most densely mapped marsupial genome.

### Comparative analysis of gene arrangement between wallaby and opossum

**Figure 6:**
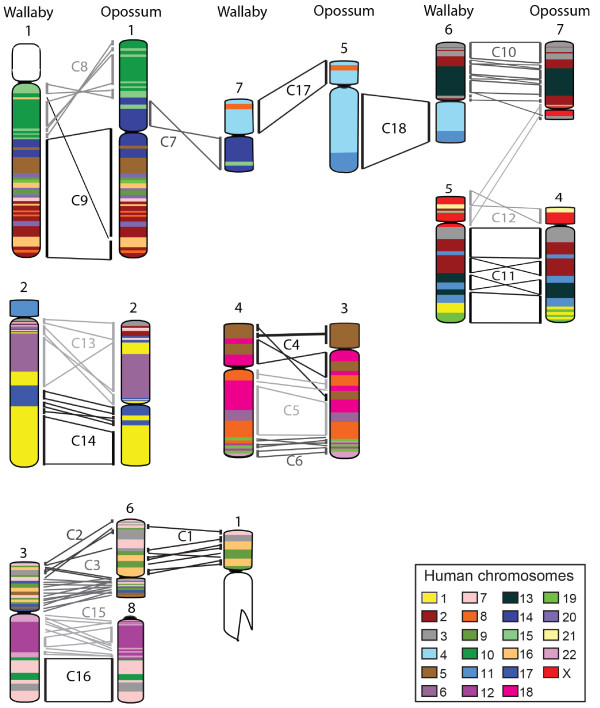
**Comparative maps of wallaby and opossum chromosomes.** Conserved gene blocks are indicated by bars alongside chromosomes and their orientation shown by lines linking bars from the two species. The conserved segment identified from chromosome painting [6] to which each gene block belongs is indicated. Wallaby and opossum chromosomes have been colour-coded to reflect homology with human chromosomes.

Previous studies characterising marsupial chromosomes based on morphology [8], G-banding [7] and chromosome painting [6,14,15] report very few rearrangements between even distantly related marsupials. Our detailed cytogenetic maps of each wallaby chromosome permit a more accurate assessment of the extent of rearrangement between wallaby and opossum chromosomes. Comparative maps of each wallaby chromosome were constructed by comparing gene blocks on wallaby chromosomes with their location in the opossum genome assembly, uncovering many intrachromosomal rearrangements undetected by less sensitive cytogenetic techniques (Figure 6).

Chromosome painting, using chromosome-specific probes from the marsupial species with the highest chromosome number (*Aepyprymnus rufescens,* the rufous bettong) has previously shown that marsupial autosomes consist of 18 segments (referred to as C1 to C18) [6] conserved between all marsupial families. We were able to determine the boundaries of most of these conserved segments using our mapping data. Since the probes used for the delineation of these conserved segments were derived from *A. rufescens* (an Australian macropodiformes species, more closely related to the tammar wallaby than the opossum), the conserved segment boundaries may not reflect the gene arrangement observed in the ancestral marsupial as there may have been rearrangements that have occurred specifically in the macropodiformes lineage.

The boundaries of C4, C7 - C9, C11, C13, C14, C17 and C18 were easily elucidated from the wallaby/opossum comparative map. For example, C7 makes up an entire arm of wallaby chromosome 7, yet lies between C8 and C9 on opossum chromosome 1, making it easy to delineate the boundary of C7 on the opossum chromosome, and hence, the boundary between C8 and C9 on wallaby chromosome 1. Other boundaries were more difficult to delineate. For instance, chromosome painting shows that C1 corresponds to the short arm of wallaby chromosome 1, but genes from this segment do not lie in one discreet block on opossum chromosome 6; two genes (*RAMP3* and *PRKCD*) are at the distal to end of opossum chromosome 6 flanked by C2 genes, and *ACO1* lies amongst C3 genes. Similarly, the multiple rearrangements of segments C2 and C3 between wallaby and opossum make it difficult to conclusively place genes *RSP6, QPRT* and *DMRT1* into either segment (Figure 6). Several boundaries were difficult to distinguish due to rearrangement of two or more segments on one chromosome in both wallaby and opossum (e.g. C4, C5 and C6 on wallaby chromosome 4 and C15 and C16 on the long arm of wallaby chromosome 3).

Identification of regions of homology between wallaby and opossum using the wallaby physical map and the opossum genome assembly showed that inversions and other rearrangements fragmented the number of conserved segments from the 18 detected by chromosome painting to 76. Every chromosome displays some degree of rearrangement between wallaby and opossum (Figure 6). The largest conserved segment (corresponding to C9) lies on the long arm of wallaby chromosome 1, although *CBFA2T3* and *GALNS* within this conserved region have transposed to a different position on wallaby chromosome 1. These two genes are part of a conserved block of human chromosome 16 genes, flanked by *WWOX* and *DHX38* in opossum, implying that the opossum arrangement is ancestral and the wallaby arrangement derived. Regions showing no rearrangement between wallaby and opossum include the entire C17 and C18 regions on wallaby chromosome 7, and C16 on wallaby terminal 3q. However, chromosome 3 also boasts the most rearranged segments, with genes within segments C2, C3 and C15 displaying a very different order between the two marsupials (Figure 6), implying many intrachromosomal rearrangements.

### Reconstruction of the ancestral therian and marsupial karyotypes

We reconstructed a putative therian ancestral karyotype from which both marsupials and eutherians diverged by comparing mapping data from the wallaby and the opossum genome assembly with the vast amount of information from eutherian genome assemblies and comparative cytogenetic studies, using the chicken genome as an outgroup. We were unable to use monotremes (the most basal mammals) as an outgroup because the platypus genome assembly is so fragmented and only a small portion of the genome is anchored to chromosomes [39]. Since both the wallaby and opossum have derived diploid numbers, we used the predicted arrangement of the conserved segments for *Didelphis marsupialis* (common opossum) to represent species with a 2n = 22 karyotype, and cross-species chromosome painting data for the dasyurid *Sminthopsis crassicaudata* (fat-tailed dunnart) to represent a 2n = 14 karyotype [6], in order to determine which species has a more ancestral arrangement of the 19 conserved segments based on comparative mapping analysis (see Additional file 2 for phylogenetic tree and arrangement of conserved segments in these species).

### Example of reconstruction with segments C10, C11, and C12

We started this analysis by examining conserved segments that span large regions on just a few chicken chromosomes, in the expectation that their evolutionary history would be easier to elucidate. For instance, genes from segments C10, C11 and C12, lie on chicken chromosome 1, and additional genes from C10 and C11 are on chicken chromosomes 7, 9 and 24.

Chromosome painting has shown different combinations of these segments across different marsupial taxa [6], making it difficult to discern their ancestral arrangement. These segments have been assigned to wallaby chromosomes 5 (C11 and C12) and 6 (C10) and opossum chromosomes 4 (C11) and 7 (C10 and C12). *D. marsupialis* has a similar arrangement to *M.domestica,* but the fat-tailed dunnart has all three segments fused in the order C10, C12 and C11 to form chromosome 3 [6,14]. The arrangement of these three segments is thus different in 2n = 22 and 2n = 14 species.

**Figure 7:**
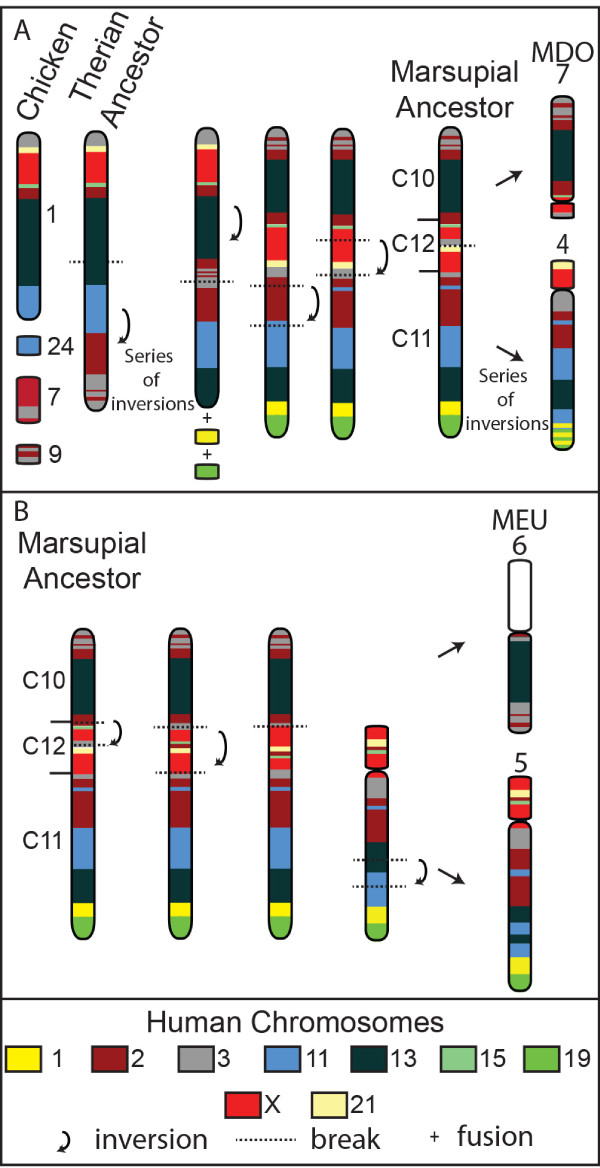
**The predicted ancestral therian chromosome containing segments C10, C11 and C12 and the derivation of opossum and wallaby chromosomes. (A)** The predicted therian ancestral chromosome aligned against chicken chromosomes containing C10, C11 and C12 genes. An inversion and the addition of genes corresponding to part of human chromosomes 1 and 19 to the distal end of this chromosome and two more inversion events result in a putative marsupial ancestral chromosome consisting of all three segments in the order of C10, C12 and C11. Opossum (MDO) chromosomes 4 and 7 are derived from a fission event taking place in segment C12. **(B)** Wallaby (MEU) chromosomes 5 and 6 are derived from the predicted marsupial ancestor via inversions, a fission between C10 and C12 and a further inversion within C11.

The ancestral arrangement of these three conserved segments is easily reconstructed by comparing the chicken gene arrangement with that in the two marsupials (Figure 7). The ancestral therian chromosome, consisting of segments C10, C12, C11, can be easily derived by adding genes from chicken chromosomes 7 (HSA2 and 3 genes), 9 (HSA2 and 3) and 24 (HSA11) to the distal end of chicken chromosome 1. By using the chicken gene order as a guide, the marsupial ancestral chromosome could have been formed by two large and two smaller inversions, resulting in the ancestral arrangement C10-C12-C11. Opossum chromosomes 4 and 7 would be the result of a fission event between HSA3 and 21 genes (Figure 7A). Wallaby chromosomes 5 and 6 are also easily derived from our predicted ancestral chromosome, with two inversions rearranging genes in C12 and fission separating C10 from C12 (Figure 7B). Subsequent intrachromosomal rearrangements, occurring after wallaby/opossum divergence, account for the current arrangement of C11 genes in these two species.

There is an association of genes from HSA3 and 21 on the marsupial ancestral chromosome reconstructed above. An association of these genes has been found by various methods in many eutherian genomes, and was proposed to have been present in the boreoeutherian ancestor [40]. The failure to observe this association in the opossum genome assembly challenged this hypothesis: however, we now see that it was, indeed ancestral to marsupials as well as eutherians, and HSA3 and HSA21 underwent fission independently in the opossum [41]. This synteny group has also been independently disrupted in the wallaby by an inversion. In fact, the region surrounding C12, consisting of genes from human chromosomes 2, 3, 15, 21 and the added region of the X has been a hotspot for inversions (Figure 7), with several breakpoints apparently reused during marsupial evolution. It is also noteworthy that this region contains the sites of centromeres in wallaby and opossum. Breakpoint reuse coinciding with positions of centromeres or latent centromeres has been reported for the karyotypically diverse Macropodidae family [42], and may be a more common feature of chromosome restructuring across marsupials.

### Reconstruction of all other segments

By employing the same approach used to reconstruct the ancestral arrangement of segments C10 to C12, we have been able to determine the most likely arrangement of the other conserved segments in the ancestral marsupial, and therian mammals.

**Figure 8:**
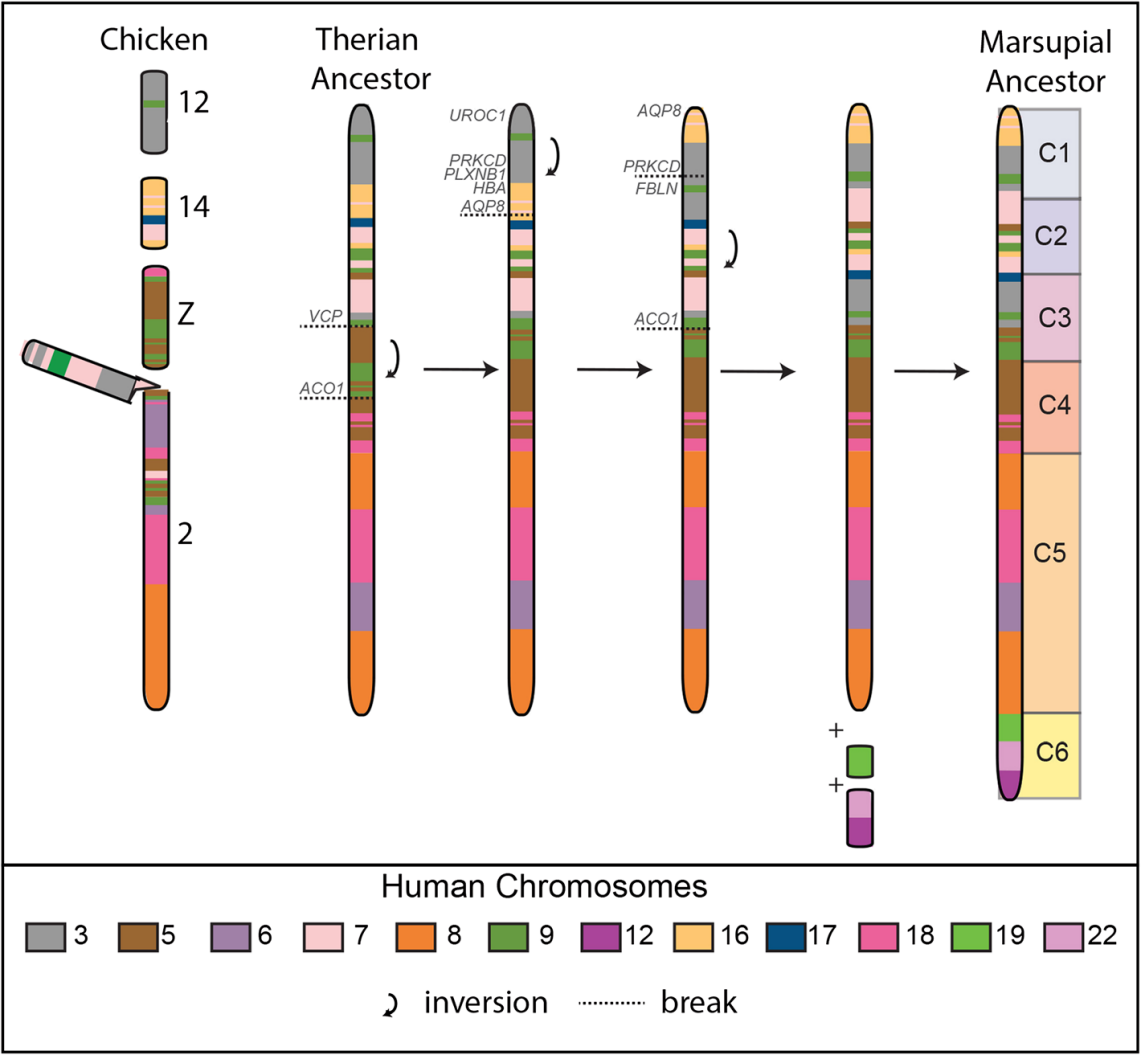
**Derivation of ancestral marsupial chromosome consisting of segments C1 to C6.** The predicted therian ancestral chromosome containing segments C1-C5 essentially corresponds to four chicken chromosomes: 12, 14, Z and a large portion of chromosome 2. Inversions and addition of chromosomal segments corresponding to human chromosomes 19, 12 and 22 to the ancestral therian chromosome ultimately led to the formation of the ancestral marsupial chromosome 1.

**Figure 9:**
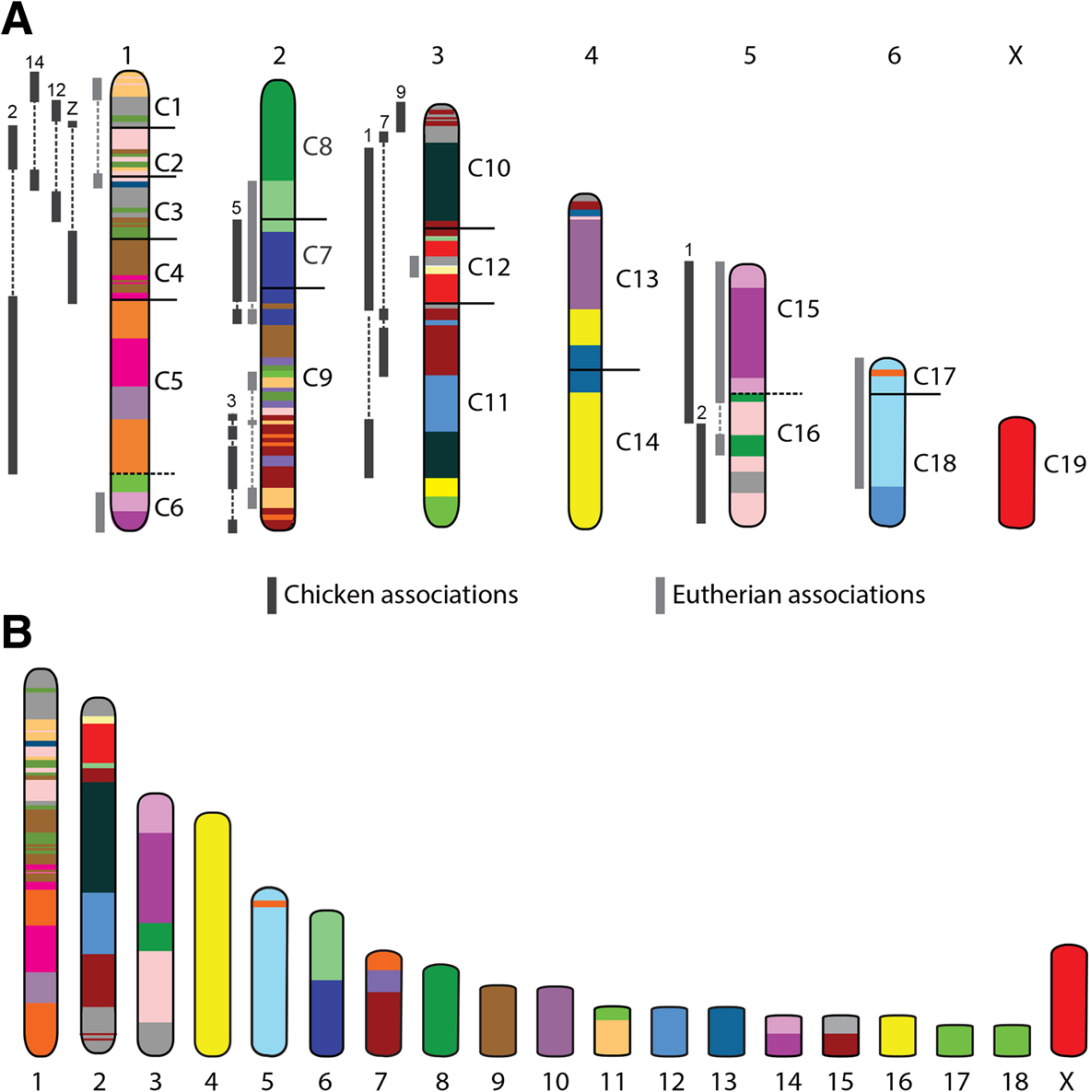
**Predicted ancestral marsupial and therian karyotypes. (A)** The 2n = 14 ancestral marsupial karyotype, predicted based on comparative mapping data, are colour-coded to show homology to human chromosomes (same colour-code as shown in Figure 6). Segments from different human chromosomes with known associations in eutherians (light grey) indicated to the left of the chromosomes. Associations of genes in chicken are indicated in dark grey with the number of the chicken chromosome shown above. Dotted lines indicate blocks from the same chicken or ancestral eutherian chromosome. **(B)** The predicted 2n = 19 therian ancestral karyotype. Chromosomes have been colour-coded to reflect homology with human chromosomes (refer to key in Figure 6).

Different combinations of segments C1 to C6 are observed in different marsupial species, with segments C4-C5-C6 forming chromosome 1 in *D.marsupialis* and chromosome 8 in this species consisting of segments C3 - C1 - C2. In *S.crassicaudata,* all six segments are joined in the order C2-C1a-C4a-C3-C1b-C4b-C5-C6. Comparative analysis of gene arrangement on these segments provides evidence that many of the genes from these six segments were probably part of a single block of genes in the therian ancestor. In chicken, genes from all six segments are found predominantly on chromosome 2 (corresponding to HSA3, 5, 7, 8, 9 and 18), the Z chromosome (corresponding to HSA5, 9 and 18), chromosome 12 (HSA3 and 9) and chromosome 14 (HSA7, 16 and 17). Genes from the chicken Z chromosome map to three segments (C1, C3, C4) in the wallaby and chromosomes 5, 8, 9 and 18 in human (Figure 8 and Figure 9A), providing a particularly important piece of evidence linking C1 - C3 with C4 in the therian ancestor. It appears that the fusion of genes from chromosomes 2 and Z occurred early in the evolution of therian mammals (Figure 9). This fusion event was probably followed by two additional fusions of genes corresponding to chicken chromosomes 12 and 14 and a series of inversions to give rise to the ancestral marsupial chromosome consisting of segments C1 to C6. Thus, *D.marsupialis* has a derived arrangement arising from a fission between C3 and C4.

Segment C9 makes up the entire *D. marsupialis* chromosome 2, but is combined with C1 and C8 in the wallaby to form chromosome 1, and C7 and C8 on opossum chromosome 1. A comparison of the arrangement of genes on chromosome 1 in opossum and wallaby clearly shows a highly conserved C9 region on the long arm shared by both species, and a few inversion events within C8 separating the species (Figure 6). In the opossum, these two segments are separated by C7, a segment that is located on chromosome 7 in the wallaby. This segment consists of human chromosome 14 genes, which also extend into C9, and human chromosome 15 genes that extend into C8. The association of human chromosome 14 and 15 genes has also been observed in many eutherian taxa and has been proposed to represent the ancestral eutherian arrangement [41]. Comparisons of arrangement between genes mapped from this region in wallaby with the location of orthologues in chicken, opossum, cow, macaque and human clearly show that human chromosome 14 and 15 genes would have been part of one chromosome segment in the therian ancestor, with genes from C9 intermingled with genes from C7 in chicken and the eutherian mammals (Additional file 3). Hence, the arrangement of C8, C7 and C9 together on one chromosome, as observed on opossum chromosome 1 and in species with a 2n = 14 karyotype, probably resembles their arrangement on the ancestral marsupial chromosome (Figure 9A).

This reconstruction specifically contradicts the proposal that the segments were originally separate in a 2n = 22 marsupial ancestor and were united by centric fusion in 2n = 14 species. An important piece of evidence for the hypothesis of an ancestral marsupial chromosome number of 22 was the observation by Svartman and Vianna-Morgante [12] of interstitial telomere signals in the pericentric region of opossum chromosome 1, near the junction of C7 and C9, which they interpreted as evidence of a centric fusion event. However, an alternative explanation is that the interstitial signals represent satellite DNA [43,44], and later C-banding experiments showed that the interstitial signals on opossum chromosome 1 do coincide with pericentric heterochromatin, and hence are not evidence of a past fusion event [21]. The comparative mapping data presented above supports this view. Moreover, it has become clear by observing the location of interstitial telomere signals on marsupial chromosome homology maps that many of these signals are not located at sites where past fusion events would have occurred. For instance, interstitial signals are present on chromosome 6 in *Sminthopsis crassicauda* (Additional file 2), a chromosome which would not have undergone fusion from either a 2n = 14 or 2n = 22 ancestor. Instead, these signals may actually be the remnants of inversions involving telomeric sequence [45].

Segments C13 and C14 are joined in most marsupial species, with the notable exceptions of *D. marsupialis* and the brushtail possum (*Trichosurus vulpecula*). In both the wallaby and opossum, chromosome 2 consists of segments C13 and C14. Comparative maps support the hypothesis that these two segments were also joined in the marsupial ancestor. For instance, genes from human chromosome 17 are in both C13 and C14, and these genes are intermingled on chicken chromosomes 18 and 19 (Additional file 4), indicating that these genes were part of a single chromosome in the therian ancestor (Figure 9B). It is less parsimonious to propose that the separation of these two segments seen in *D. marsupialis* and *T. vulpecula* represents an ancestral marsupial state, as that would require these regions to fuse in the therian ancestor, then split in the marsupial ancestor, only to fuse again to produce the arrangement observed in most marsupial species.

Segments C15 and C16 are adjacent in all marsupials examined so far, except *A. rufescens*, the marsupial with the highest diploid number of 2n = 32. The separation of these two segments is therefore assumed to be the result of a fission event specific to this species. In eutherian mammals, these genes are spread across several chromosomes but genes from both segments are found intermingled on bovine chromosomes 4 and 13 and human chromosomes 7 and 10 (Additional file 5), suggesting that segments C15 and C16 were together prior to the divergence of therian mammals. Further support for the combination of these two segments in the therian ancestor comes from synteny group association of human chromosomes 10p, 12pq and 22qt predicted to have been present in the boreoeutherian ancestor [46]. Outgroup analysis reveals that C15 and C16 genes lie in a block on chicken chromosome 1, and other C16 genes lie on chicken chromosome 2 (Figure 9A). Froenicke et al. [46] referred to the association of HSA10p and 12pq as weak, but finding these regions combined on the one chromosome in marsupials suggests that it was actually present prior to the divergence of therian mammals.

In all except the macropodiformes species (*M. eugenii* and *A. rufescens*), segments C17 and C18 are fused, which is presumably the ancestral arrangement of these two segments. Genes spanning both segments are found on chicken chromosome 4 (Figure 9A) and an association has also been observed in eutherians (corresponding to HSA4/8p) [40].

### The predicted therian ancestral karyotype

We reconstructed the putative therian ancestral karyotype based on the associations of chromosome segments we observed in marsupials, the known associations in eutherians [40] and by comparison to chicken as an outgroup. The reconstructed karyotype consists of 19 chromosomes, including three large chromosomes that are very similar to the predicted ancestral marsupial chromosomes 1, 3 and 5. Comparisons with the most basal mammals, the monotremes, could have provided additional insight for the reconstruction of the therian ancestor but the fragmented nature of the genome assembly has made such a comparison difficult at this time [39]. For instance, we have defined the therian chromosome 2 in Figure 7A as not including HSA1 and HSA19 genes present in the predicted marsupial ancestral chromosome 3 because there was no evidence from the genomes included in this study that this would be the case. The platypus genome could have more definitively resolved this issue but genes from these two human chromosomes are assembled into many contigs and ultracontigs in the platypus genome assembly. Similarly, an alternative therian karyotype could consist of 2n = 18 chromosomes, where genes corresponding to HSA19 are distributed between just two chromosomes rather than the three we predicted based on the distribution of these genes in the wallaby and opossum genomes. Of course, it is possible that a fission event separated these genes in the marsupial lineage, meaning that they were together in the therian ancestor. As previously mentioned, HSA19 genes in the platypus genome assembly have been assigned to many contigs and ultracontigs.

**Figure 10:**
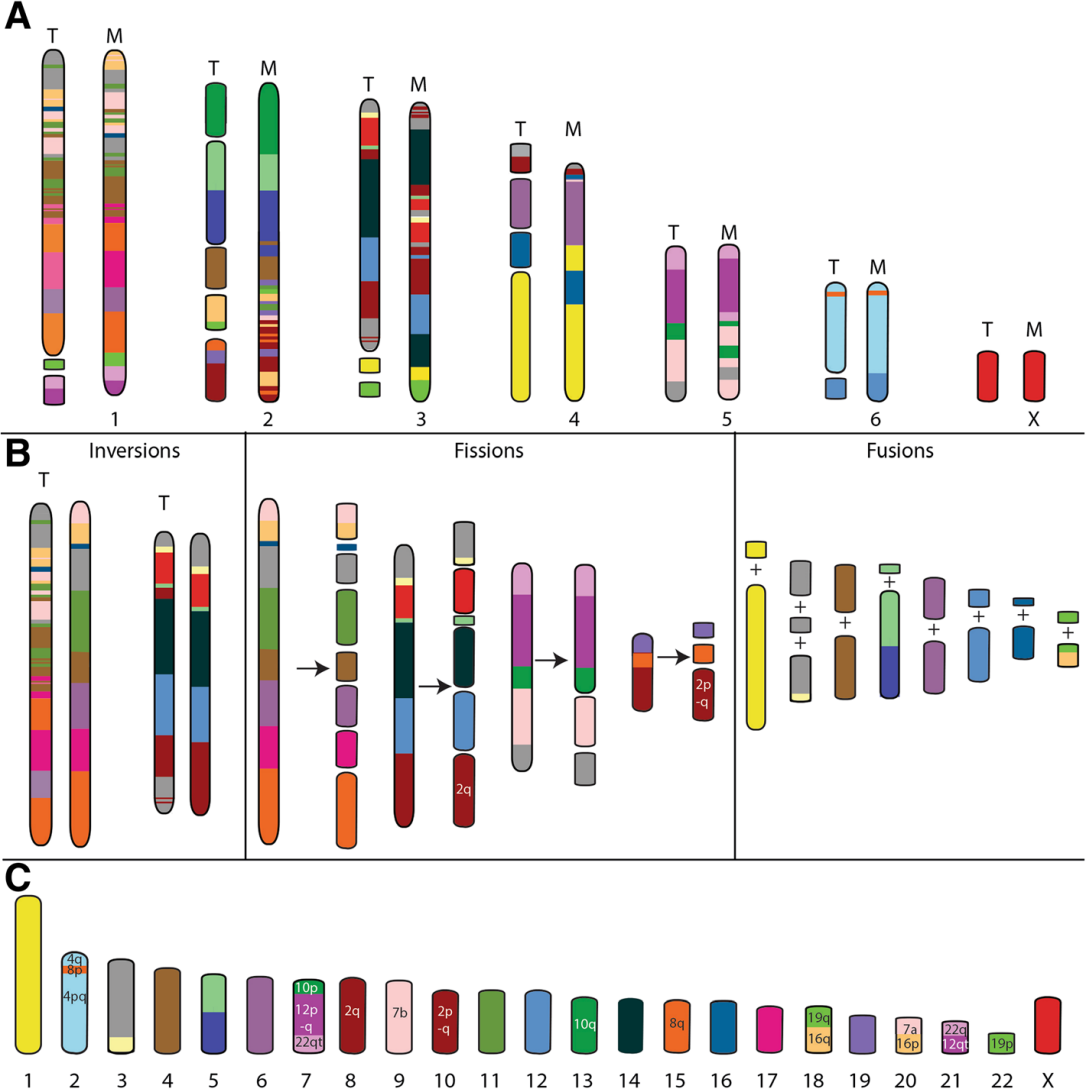
**Derivation of (A) marsupial and (B,C) eutherian ancestral karyotypes from the predicted ancestral therian karyotype. (A)** The predicted ancestral marsupial karyotype was formed by fusions of the predicted therian chromosomes. **(B)** Inversions, fusions and fissions led to **(C)** the previously predicted ancestral eutherian karyotype [40]. T – Therian, M- Marsupial. Chromosomes have been colour-coded to reflect homology with human chromosomes (refer to key in Figure 6).

A 2n = 14 ancestral marsupial karyotype is very simply derived from fusions of the predicted therian chromosomes, followed by inversions (Figure 10A). The putative eutherian ancestral karyotype previously predicted from cross species chromosome painting [40] or a combination of cytogenetic and genome sequence analysis [47] can also be easily reconstructed from these predicted therian chromosomes by a series of inversions, fissions and fusions (Figure 10B and C). Fissions appear to have featured prominently in chromosome evolution leading to the eutherian radiation whereas fusion of chromosomes has led to the larger chromosomes of marsupials.

It is important to bear in mind that there are some limitations associated with any ancestral karyotype reconstruction, as the process relies on the data available for representative extant species. We have already alluded to how a well-assembled and anchored platypus genome could have improved the confidence of our predictions. In addition, there are only two marsupials species with a gene map of sufficient density or an anchored and oriented genome assembly. Furthermore, a limitation of the physical map of the wallaby genome is that it provides information only for the location of the large blocks of conserved genes identified between comparisons of the opossum and human genome, resulting in genes from conserved synteny blocks smaller than the 100 kb block size limit of the Ensemble Synteny Viewer [31] being excluded from our analysis. For efficient mapping of the genome by FISH, we focused on mapping large blocks of genes that did not necessarily have a conserved gene order between opossum and human, meaning that there may be additional rearrangements that have gone undetected in comparisons between the wallaby and other genomes. In addition, we have used chicken as a guide for gene order when reconstructing the events leading to the gene arrangement observed in extant marsupials. The chicken gene order may not represent the gene order of the amniote ancestor and hence, may not accurately reflect the gene arrangement in the therian ancestor. This in turn could impact on the predicted therian and marsupial ancestral karyotypes. Nonetheless, our ancestral karyotype reconstructions provide a basis for more detailed analysis if additional anchored marsupial and/or monotreme genomes become available in the future.

## Conclusions

The debate over the whether the ancestral marsupial karyotype was 2n = 14 or 2n = 22 has persisted for many years because data were not available to compare the marsupial chromosome arrangement with that observed in representatives of other lineages. Our dense physical map of the tammar wallaby genome and the anchored assembly of the opossum genome have allowed us to address this issue. Our analysis has enabled us to construct a marsupial ancestral karyotype, providing further support for a 2n = 14 ancestral marsupial karyotype. Comparative mapping data suggests that inversions have played a major role in shaping marsupial genomes. Furthermore, comparisons with eutherian and chicken genomes have given substantial insight into the evolution of mammalian genomes, having enabled us to predict the chromosome complement of 2n = 19 for the therian ancestor. These chromosomes underwent fusion leading to the marsupial lineage and fission leading to the eutherian ancestor. We are now a step closer to determining the karyotype of the ancestor for all mammals. Understanding how genome arrangement has changed over time may ultimately help us to elucidate the genome changes, and their consequences on gene regulation and function, that have led to the major phenotypic differences observed between the different mammalian lineages.

## Methods

### Mapping of evolutionary conserved blocks

Large blocks of genes conserved between human and opossum were identified using the Ensembl Synteny Viewer tool [31]. The same approach for BAC library screening and FISH mapping was taken as described in Deakin et al. [30]. Briefly, overgo probes (see supplementary material for overgo information) were designed for genes at the ends of conserved blocks (or for one gene for blocks smaller than 3 Mb) using the Overgo Maker program developed by The Genome Institute at Washington University. Specificity of the resulting 40 bp probe was verified by BLAST searching the MonDom5 assembly as well as the wallaby sequence trace archives. Pools of up to 40 pairs of overgos were used to screen the male wallaby BAC library (Me_KBa; Arizona Genome Institute, USA). BACs isolated from library screening were subjected to a second round of screening via dot blots in order to determine which BACs were positive for each gene. Two-colour FISH was used to orient conserved blocks on male metaphase chromosomes, with BACs directly labelled with either Orange or Green dUTP (Abbott Molecular Inc., Des Plaines, IL, USA), hybridised to metaphase chromosomes and images of hybridisation signals captured as described in Deakin et al. [30].

#### Comparative map construction

Comparative maps were constructed by extracting data from assembled genomes, mainly opossum, chicken and human, using the Ensembl Biomart tool [48] and comparing gene order between species using AutoGRAPH synteny visualisation tool [49], with manual input of tammar wallaby gene mapping data.

## Abbreviations

BAC: Bacterial articifical chromosome; FISH: Fluorescent in situ hybridisation; HSA: Homo sapiens; Kb: Kilobase; Mb: Megabase; MEU: *Macropus eugenii*; MDO: *Monodelphis domestica.*

## Competing interests

The authors declare that they have no competing interests.

## Author contributions

JED, and JAMG designed the study. JED, MLD, EK, and VSP designed overgo probes and screened the BAC library. JED, EK, AEA and NH performed FISH experiments. JED, MLD, EK, and CW analysed data and constructed comparative maps. JED reconstructed the ancestral marsupial and therian karyotypes. JED and JAMG drafted the manuscript. All authors commented on and approved the final manuscript.

## Supplementary Material

Additional file 1 Genes mapped to wallaby chromosomes, overgo sequences and the corresponding BACs. Click here for file

Additional file 2 **Arrangement of conserved chromosome segments in *Macropus eugenii, Aepyprymnus rufescens, Trichosurus vulpecula, Sminthopsis crassicaudata *** **and *Monodelphis domestica *** **as determined by chromosome painting **[6]**, and *Didelphis marsupialis *** **(predicted based on G-banded karyotype **[13]**,**[16]**).** Click here for file

Additional file 3 Arrangement of genes from segments C7 to C9 between chicken, wallaby, opossum, cow, macaque and human. Click here for file

Additional file 4 A comparison of the arrangement of human chromosome 17 genes from segments C13 and C14 between chicken, wallaby, opossum and human. Click here for file

Additional file 5 **Arrangement of genes from segments C15 and C16 genes between chicken, wallaby, opossum, cow and human.** Genes from the predicted boreoeutherian associated segments 10p + 12pq + 22qt are highlighted. Click here for file
