# Supplementary figures and images for: Reconstruction of the ancestral marsupial karyotype from comparative gene maps
Source: BMC Evol Biol. 2013 Nov 21;13:258. doi: 10.1186/1471-2148-13-258 (PMC4222502; doi:10.1186/1471-2148-13-258)

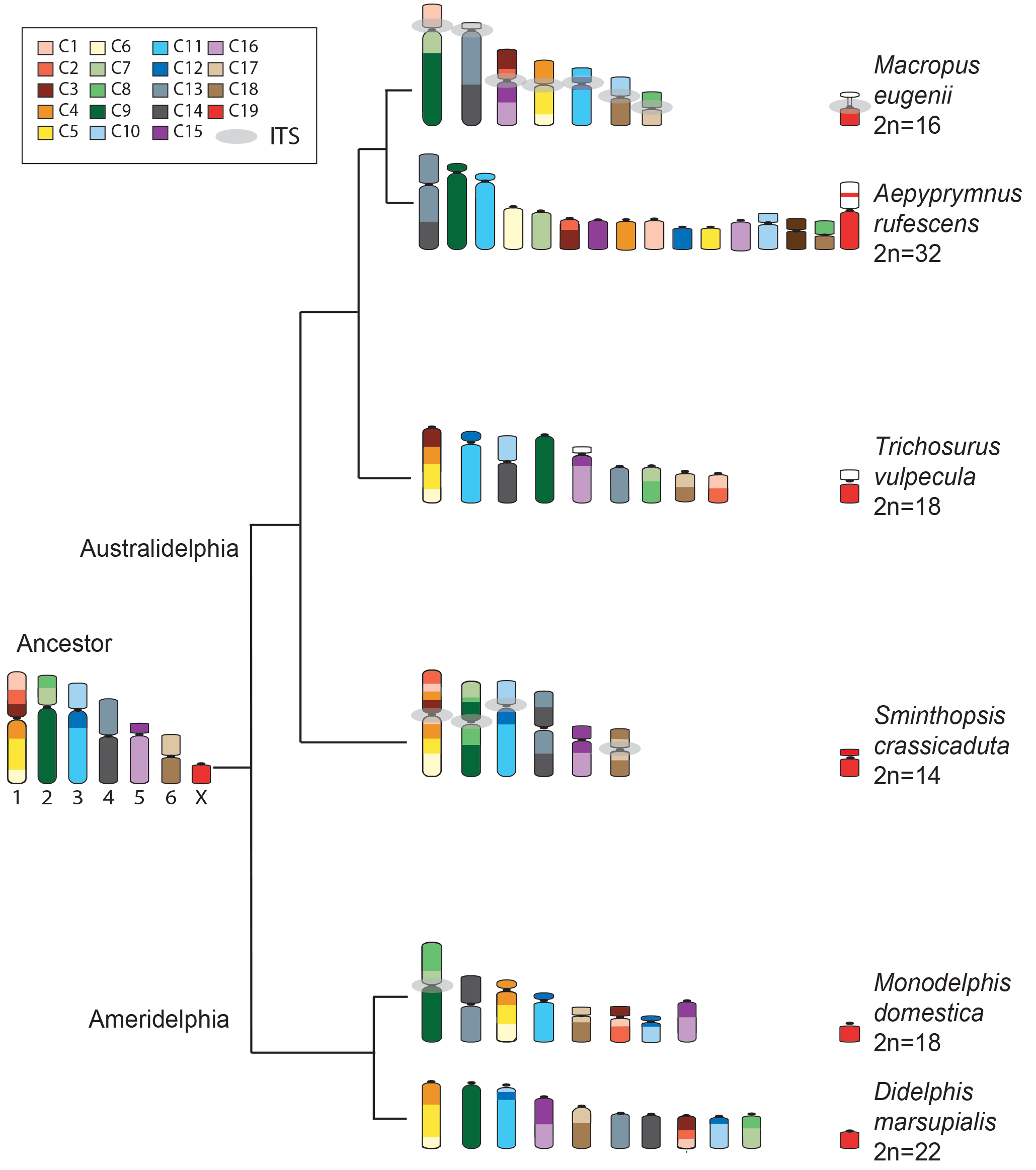

Supplement: Additional file 2 — Arrangement of conserved chromosome segments in Macropus eugenii, Aepyprymnus rufescens, Trichosurus vulpecula, Sminthopsis crassicaudata and Monodelphis domestica as determined by chromosome painting [6], and Didelphis marsupialis (predicted based on G-banded karyotype [13],[16]). [file 1471-2148-13-258-S2.tiff]

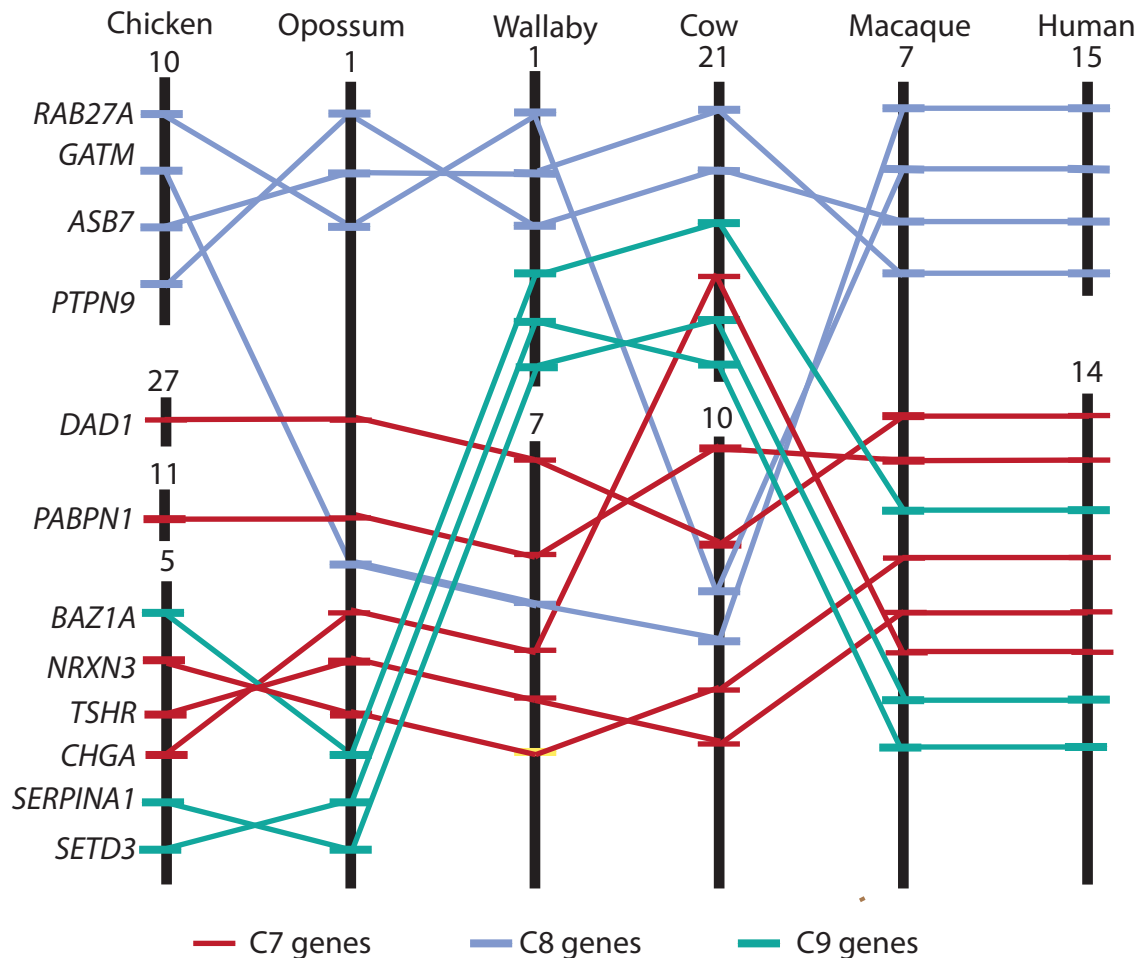

Supplement: Additional file 3 — Arrangement of genes from segments C7 to C9 between chicken, wallaby, opossum, cow, macaque and human. [file 1471-2148-13-258-S3.pdf]

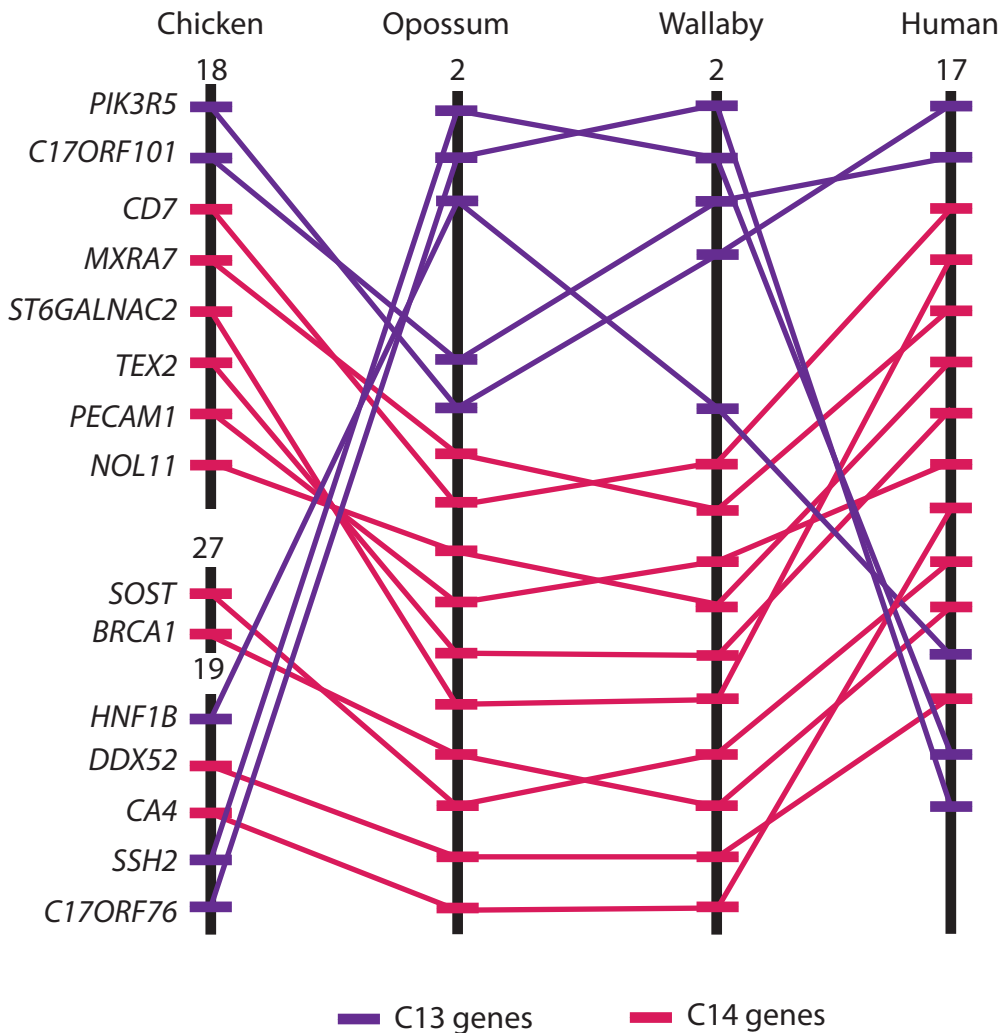

Supplement: Additional file 4 — A comparison of the arrangement of human chromosome 17 genes from segments C13 and C14 between chicken, wallaby, opossum and human. [file 1471-2148-13-258-S4.pdf]

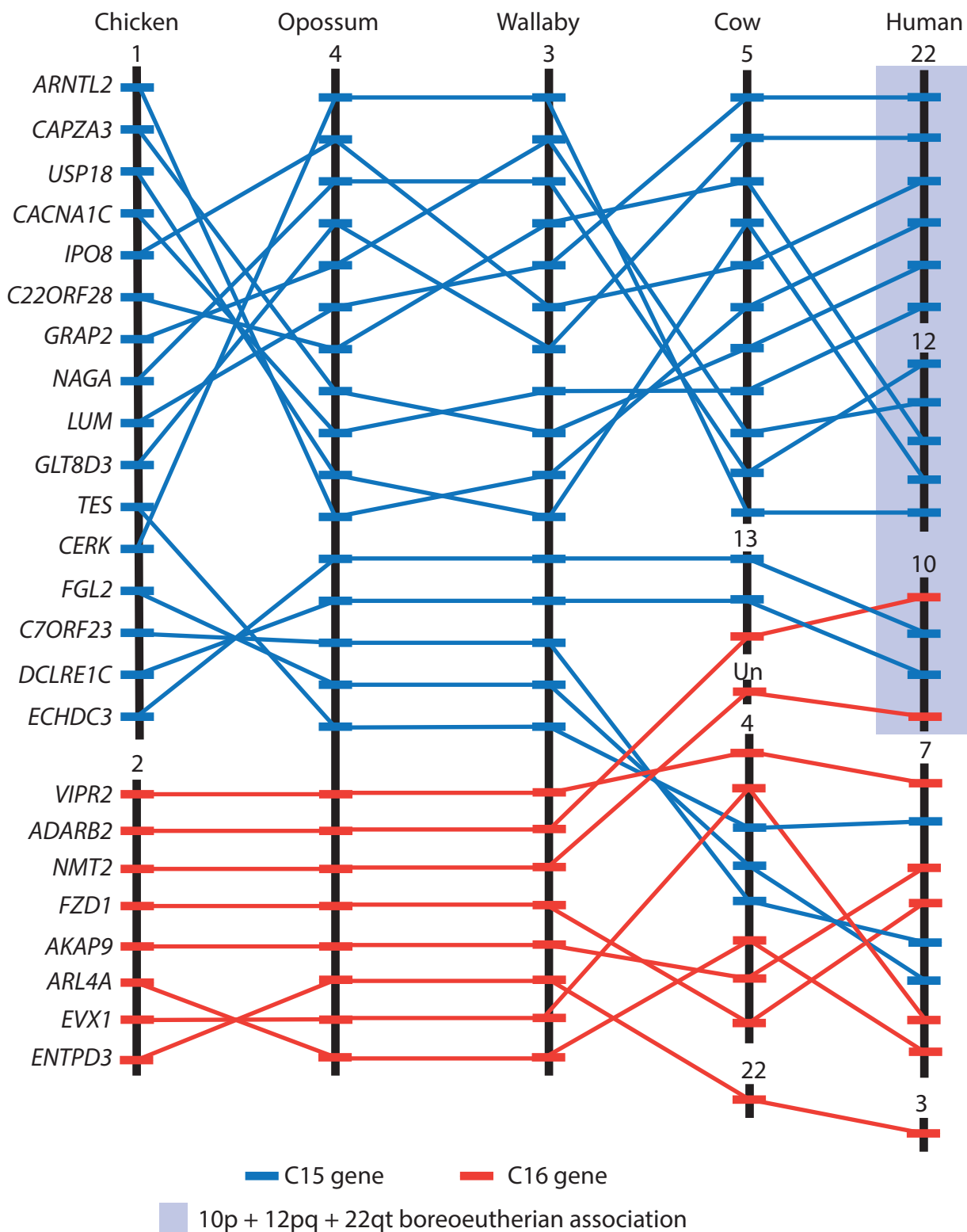

Supplement: Additional file 5 — Arrangement of genes from segments C15 and C16 genes between chicken, wallaby, opossum, cow and human. Genes from the predicted boreoeutherian associated segments 10p + 12pq + 22qt are highlighted. [file 1471-2148-13-258-S5.pdf]
